# A novel feature extraction method based on dynamic handwriting for Parkinson’s disease detection

**DOI:** 10.1371/journal.pone.0318021

**Published:** 2025-01-24

**Authors:** Huimin Lu, Guolian Qi, Dalong Wu, Chenglin Lin, Songzhe Ma, Yingqi Shi, Han Xue

**Affiliations:** 1 School of Computer Science and Engineering, Changchun University of Technology, Changchun, Jilin, China; 2 Affiliated Hospital to Changchun University of Chinese Medicine, Changchun, Jilin, China; 3 Jilin Provincial Smart Health Joint Innovation Laboratory for the New Generation of AI, Changchun Univerity of Technology, Changchun, Jilin, China; 4 Changchun University of Chinese Medicine, Changchun, Jilin, China; Federal University of Paraiba, BRAZIL

## Abstract

Parkinson’s disease (PD) is a common disease of the elderly. Given the easy accessibility of handwriting samples, many researchers have proposed handwriting-based detection methods for Parkinson’s disease. Extracting more discriminative features from handwriting is an important step. Although many features have been proposed in previous researches, the insight analysis of the combination of handwriting’s kinematic, pressure, and angle dynamic features is lacking. Moreover, most existing feature is incompletely represented, with feature information lost. Therefore, to solve the above problems, a new feature extraction approach for PD detection is proposed using handwriting. First, built on the kinematic, pressure, and angle dynamic features, we propose a moment feature by composed these three types of features, an overall representation of these three types of features information. Then, we proposed a feature extraction method to extract time-frequency-based statistical (TF-ST) features from dynamic handwriting features in terms of their temporal and frequency characteristics. Finally, we proposed an escape Coati Optimization Algorithm (eCOA) for global optimization to enhance classification performance. Self-constructed and public datasets are used to verify the proposed method’s effectiveness respectively. The experimental results showed an accuracy of 97.95% and 98.67%, a sensitivity of 98.15% (average) and 97.78%, a specificity of 99.17% (average) and 100%, and an AUC of 98.66% (average) and 98.89%. The code is available at https://github.com/dreamhcy/MLforPD.

## Introduction

Parkinson’s disease (PD) develops rapidly and usually occurs in elders [1]. Over the past two decades, the disability and mortality caused by PD have more than doubled, imposing a heavy burden globally [2]. PD leads to various movement disorders, such as rigidity, tremor, and bradykinesia [3]. Currently, there is no cure for PD, and as time goes on, PD symptoms gradually worsen and develop into disabling and difficult-to-treat symptoms [4]. Therefore, an accurate diagnosis of PD in the early stages can help diagnose and treat patients in time to slow the progression of the disease, thereby reducing the burden on patients and society. When the movement disorders of PD affect the dominant hand, patients will report dysgraphia. This is because writing is a complex process involving fine motor control, and any movement disorder will affect its quality [5]. In particular, when the fluency and coordination of hand movements decrease, it can lead to patients gradually writing smaller and having writing difficulties. As one of the early symptoms of Parkinson’s disease patients [6], handwriting has been used to diagnose PD at an early stage. Using pen and paper, physicians can detect dysgraphia and thus determine whether a patient has PD [5]. Compared with other methods, handwriting detection is more convenient and less expensive. However, this traditional method relies on the physician’s experience and vision, which is highly subjective and prone to misdiagnosis. In particular, dysgraphia is not evident in the early stages of the disease. Computer-aided diagnosis systems automatically analyze handwriting characteristics using data-driven algorithms, which can eliminate human interference and quantify and standardize handwriting analysis. Therefore, the development of computer-aided diagnosis systems helps overcome the limitations of traditional methods. Automating the detection of PD through computer systems can provide more standardized and reliable diagnostic results and help detect PD in areas with resource-poor physicians.

Two primary modalities in handwriting analysis are one-dimensional dynamic signals and two-dimensional static images. Handwriting signals are captured in real-time during the writing process through sensors [7, 8]. These sensors can capture multi-dimensional dynamic handwriting features, such as the speed, pressure, and angle during writing. These dynamic features can present abnormal behaviors and subtle changes in the patient’s writing process in greater detail, so researchers have focused more on dynamic signal analysis of handwriting rather than static images. However, there is a typical challenging problem in handwriting analysis—more robust feature extraction methods. Many researchers have conducted studies to address this problem. Nolazco-Flores et al. [9] extracted four types of handwriting signal features: time and kinematic features, such as displacement and duration; statistical features, such as basic statistical functions such as the mean; frequency domain features using the discrete Fourier transform; and cepstral features. Valla et al. [10] extracted the higher-order derivatives of the original signal up to the sixth order and considered the remaining three angles in addition to the tilt and azimuth. Deharab et al. [11] decomposed handwriting signals into intrinsic mode functions and calculated two new features to represent the nonlinearity and variability of handwriting. Ma et al. [12] proposed a new spatial-temporal-spectral fusion neural network that automatically extracts image features and frequency domain features obtained by transforming the image in seven ways, including the short-time Fourier transform. Zhao et al. [13] proposed a new spatio-temporal siamese neural network that uses two siamese networks composed of bidirectional memory neural networks and bidirectional memory neural networks to automatically extract temporal, spatial, and textural features of handwritten signatures.

However, despite some achievements of the above studies, some deficiencies remain: 1) they overlook the combined analysis of kinematic, pressure, and angle dynamic features of handwriting; 2) when performing time-frequency feature extraction of dynamic handwriting features, they do not consider all dynamic handwriting features, resulting in incomplete feature representation and the loss of feature information. Therefore, we propose a new handwriting-based feature extraction method for PD detection to address these shortcomings. First, we propose a moment feature that integrates kinematic, pressure, and angle dynamic features of handwriting signals, providing a novel perspective on describing dynamic handwriting signals. Second, to obtain a more comprehensive feature set, we propose a feature extraction method to separately extract time-frequency-based statistical features of kinematic, pressure, angle, and moment features to describe in detail the abnormal behavior and subtle changes in handwriting. Finally, since parameters affect the classification performance, we choose a meta-heuristic algorithm to find the optimal parameters of the model. Coati Optimization Algorithm (COA) is a newly proposed meta-heuristic algorithm with the advantages of strong optimization ability, etc., and has been applied to many practical problems [14]. However, in some optimization problems, COA may converge prematurely [15]. Therefore, we propose the eCOA algorithm to alleviate this problem. The experimental results show that the proposed method performs excellently and, as a non-invasive, low-cost, and implementation-based method, can provide more effective support for PD detection and be widely used in clinical practice.

Our main contributions of this work are as follows:

1. We propose a moment feature that comprehensively reflects the original handwriting signal’s kinematic, pressure, and angle information.
2. We propose a feature extraction method based on the kinematic, pressure, angle, and moment features of handwriting. This method extracts TF-ST features from dynamic features to more precisely describe subtle differences in handwriting.
3. We propose an eCOA optimization algorithm for global optimization to find the global optimal parameters combination and improve classification performance.
4. We conducted extensive experiments on the Cc-PhD dataset (collected by the authors) and the publicly available PaHaW dataset.

The remaining parts of this paper are as follows: The Related works section discusses the existing feature extraction methods and metaheuristic algorithms in PD detection. The Method section introduces the two datasets used in this paper (Cc-PhD and PaHaW) and the proposed methods. The Results and discussion section presents the experimental results on the two datasets and analyses the effectiveness of the proposed feature extraction methods and eCOA. Finally, the Conclusions section provides conclusions and discusses the limitations of the research and future research directions.

## Related works

### Feature extraction based on dynamic handwriting

Based on the original handwriting signals, derived feature vectors such as kinematics and pressure have been widely adopted in numerous studies, including velocity, acceleration, jerk, and pressure variations [16, 17]. Researchers mainly extract handwriting features from the original signals and derived vectors for analysis. Based on the extraction method, features can be categorized into two types: deep features and handcrafted features.

In deep feature extraction, discriminative feature representations are automatically extracted from the data using deep learning models. Ribeiro et al. [18] proposed a model based on bidirectional gated recurrent units to extract temporal features from handwriting signals. Diaz et al. [19] proposed a model based on one-dimensional convolution and bidirectional gated recurrent unit (BiGRU) to extract temporal features from raw and derived sequences automatically. Ma et al. [20] proposed a new transformer model to extract handwriting signal features while extracting the kinematic features of handwriting signals and fusing the two features to obtain the final feature set. Wang et al. [21] encoded the dynamic signal of handwriting as a two-dimensional RGB image and a three-dimensional voxelated point cloud and then applied a one-dimensional, two-dimensional, and three-dimensional CNN to extract depth features automatically. Wang et al. [22] considered the potential importance of local signal segments, segmented the handwriting signal to obtain local signal segments, and proposed a one-dimensional hybrid network LSTM-CNN to extract temporal features. Deep features can learn richer and more abstract feature representations and have better generalization capabilities. However, training these models requires a large amount of data and computing resources, and models are difficult to understand and interpret intuitively, with poor interpretability. Besides, although the extraction of depth features is simple, due to the small sample data, the model cannot learn a feature representation with strong generalization ability and is prone to overfitting.

Handcrafted features are manually designed by researchers based on domain knowledge and experience, and the design process is relatively complex. However, Handcrafted features can effectively capture relevant data information, offer high interpretability, and demand relatively low computational resources. For handcrafted features, besides extracting simple essential statistical characteristics from handwriting signals, researchers design and extract more complex features to provide a more profound understanding of handwriting. Impedovo et al. [23] applied signal processing techniques and proposed a set of velocity-dependent features. Aouraghe et al. [24] applied some filters to velocity, acceleration, and jerk kinematic feature vectors, proposing new features for handwriting kinematics in the temporal and spectral domains. Deharab et al. [25] introduced dynamic writing traces warping (DWTW) and proposed DWTW-based features in the coordinates of handwriting signals. Ammour et al. [26] used kinematic, pressure, inclination, and stroke features to construct a feature set and used correlation to select the best feature subset. Afonso et al. [27] divided signal segments through a sliding window and applied discrete wavelet transform to obtain local descriptors, extracting histogram features from the descriptors. Lamba et al. [28] utilized genetic algorithms-based mutual information gain methods to choose the most discriminative kinematic features. Kumar et al. [29] analyzed kinematic features of handwriting under different directions and pen states, extracting 24 types of kinematic features.

However, the above studies all extract features from various dynamic feature data for analysis, ignoring the interactions and connections between dynamic feature data, which leads to the failure to discover potential behavioral patterns. Moreover, not all handwriting signals are considered when extracting time-frequency features from dynamic handwriting features, inevitably resulting in the loss of feature information. Therefore, to solve the above problems, firstly, this paper proposes the moment feature, which integrates kinematic, angle, and pressure dynamic feature data to reflect the interaction of various dynamic features and thus capture the overall impact of PD on patients’ handwriting control. Secondly, a feature extraction method is proposed to extract time-frequency-based statistical features of each type of dynamic feature data separately to obtain a more comprehensive feature set, which can describe abnormal behaviors and subtle changes in handwriting in detail.

### Metaheuristic algorithms

Metaheuristic algorithms have been used to optimize parameters for Parkinson’s disease detection models, thereby improving their performance. Olivares et al. [30] optimized the parameters of the extreme learning machine for PD prediction via voice using the Bat Algorithm. El-Hasnony et al. [31] combined the Grey Wolf Optimizer and Particle Swarm Optimization to optimize an adaptive neuro-fuzzy inference system. Li et al. [32] applied metaheuristic algorithms for feature selection using the proposed improved Discrete Artificial Bee Colony Algorithm. Rajammal et al. [33] used five transfer functions to binary improve the Grey Wolf Optimizer for selecting the best feature set for PD classification. Hashim et al. [34] combined multiple techniques with the Hunger Games Search Algorithm and applied them to PD classification using Parkinson’s disease phonation datasets. Cuk et al. [35] improved the Crayfish Optimization Algorithm for PD detection. Cincovic et al. [36] proposed an improved metaheuristic algorithm for early prediction of PD using finger-tapping test data based on the Sinh Cosh Optimizer. Although many metaheuristic algorithms have been applied to Parkinson’s disease detection, according to the “No Free Lunch” theory, no algorithm is suitable for all optimization tasks. The COA algorithm has been applied to many practical problems due to its excellent search performance. However, in some issues, COA may face the problem of premature convergence. Therefore, to solve the above issues, this paper proposes an escape Coati Optimization Algorithm. By considering the prey escape probability, eCOA can explore the search space more flexibly and get rid of the local optimum.

## Method

**Fig 1:**
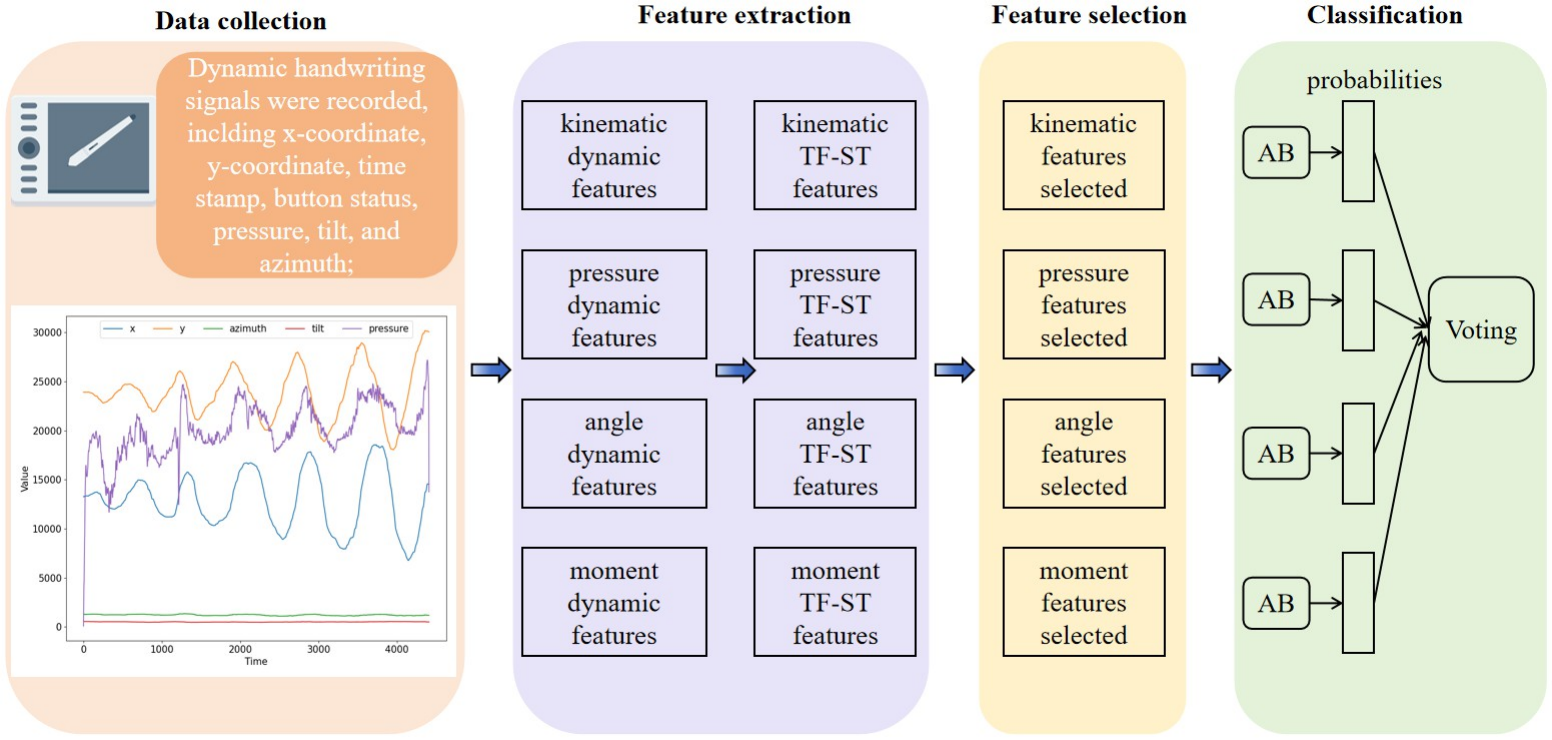
The proposed framework for PD detection. Abbreviations: TF-ST: time-frequency-based statistical. AB: adaboost.

After acquiring the dynamic handwriting signals using the digitizing tablet, four types of features are extracted, which are kinematic, pressure, angle, and moment. The proposed moment feature effectively aggregates kinematic, pressure, and angle dynamic features, providing a more comprehensive feature representation. Then, based on the four categories of features mentioned above, TF-ST features are further extracted by a new feature extraction method. These features offer finer grain and multidimensional information for the framework. Subsequently, the random forest feature selection method is employed to select the most representative features subset. The AdaBoost classifier is then employed for training and prediction, with classifier parameters optimized using the proposed eCOA algorithm. Considering the different physical and statistical meanings of each type of feature, feature extraction, training, and prediction are conducted separately for each feature type. After obtained the prediction results for each feature type, an adaptive weighted sum is performed to obtain the final prediction result, as shown in the Fig 1.

### Preliminary

In this study, the Cc-PhD and PaHaW datasets are used. The Cc-PhD dataset (collected by the authors) includes PD patients, essential tremor (ET) patients, and healthy controls (HC). Since ET also causes dysgraphia [37], ET was added to the original classes to ensure that the proposed method can accurately detect PD rather than misclassify all dysgraphia as PD. The PaHaW dataset (publicly available) includes PD patients and HC. Detailed descriptions of the two datasets are provided below.

#### Cc-PhD

The Changchun Parkinson’s disease Handwriting Dataset (Cc-PhD) is jointly collected by Affiliated Hospital to Changchun University of Chinese Medicine and Changchun University of Technology as part of a project on handwriting-based detection of PD and ET. The study is approved by the Science and Technology Ethics Committee of Changchun University of Technology (Report No. 2024009) and the Ethics Committee of Affiliated Hospital to Changchun University of Chinese Medicine (No. CCZYFYKYLL2024). Offline recruitment includes patients with PD or ET and HC, with diagnoses confirmed by experienced neurologists. Data collection began on April 12, 2024, and ended on June 30, 2024. Ninety-seven subjects participated in this study (mean age: 58.70±14.78 years, 48 males/49 females), including 31 PD patients, 31 ET patients, and 35 HC participants. All participants enrolled voluntarily and provided informed consent in writing, and the study did not involve minors. All participants are right-handed and native Chinese speakers.

The study uses a Wacom Intuos Pro digitizing tablet (338 × 219 × 8 mm) and its accompanying pressure-sensitive pen to collect handwriting data with 8192 pressure levels and 200 Hz sampling frequency. Before starting the tasks, participants are asked to perform some preliminary exercises, such as writing their names, to familiarize themselves with the digitizing tablet and the overall process. For each sample, the tablet captured seven independent dynamic features: x-coordinate, y-coordinate, timestamp, button status, pressure, tilt, and azimuth. The button status value was either 0 or 1, with 0 representing the pen in the air and 1 representing the pen touching the surface.

**Fig 2:**
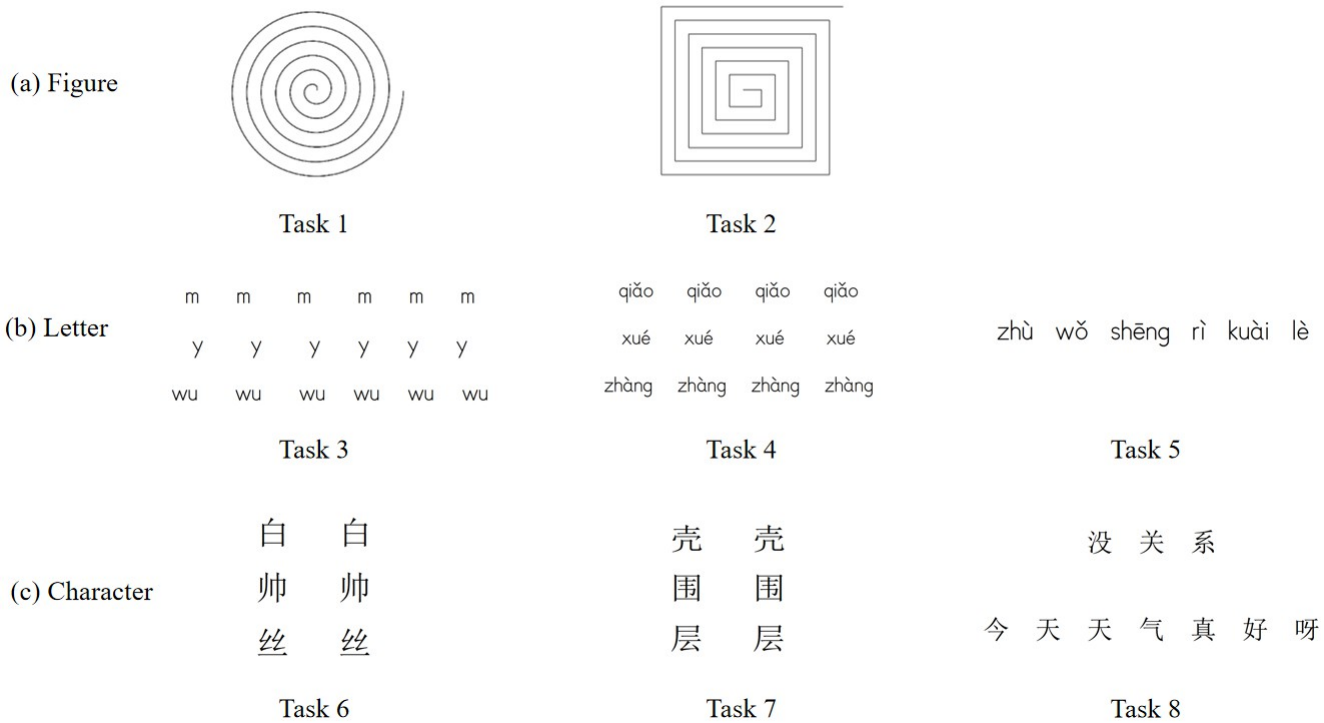
Handwriting tasks used in the Cc-PhD dataset.

**Fig 3:**
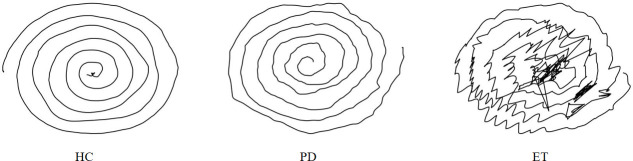
Spirals drawn by PD, ET, and HC patients.

All patients are required to complete several handwriting tasks in sequence, comprised of three categories: graphical, alphabetical, and character-based. Spiral and meander patterns are chosen as graphical tasks. The spiral task is a conventional test for tremor examination, and both spiral and meander patterns have been widely used by researchers to diagnose PD or ET [8, 38, 39]. The alphabetical and character-based tasks follow a principle of increasing complexity, starting from single letters (characters) to multiple letters (characters) and finally to whole sentence. According to the Delta-Lognormal Kinematic handwriting theory [40], letters or characters that include upward and downward strokes are selected. Additionally, the criteria of selection included more movement directions, simple spelling, and easy grammar [41]. Fig 2 provides a detailed description of the handwriting tasks used. Fig 3 shows the spiral patterns drawn by PD, ET, and HC patients.

#### PaHaW

The Parkinson’s disease handwriting (PaHaW) database was acquired with the Movement Disorders Center at the First Department of Neurology, Masaryk University, and St. Anne’s University Hospital in Brno, Czech Republic. The PaHaW dataset collected dynamic handwriting data from 75 participants (mean age: 65.83±11.56, 39 males/36 females), including 37 PD and 38 HC patients. All participants are sequentially instructed to complete eight handwriting tasks: Archimedean spiral, letters, words, and sentences. The equipment captures the same seven independent dynamic features as mentioned above during the drawing process.

### Kinematic and pressure dynamic features

Kinematic and pressure characteristics have been adopted by many handwriting studies. Kinematic features can be computed from raw signals, which include x-coordinate, y-coordinate, displacement, velocity, acceleration, and jerk. Displacement represents the distance between two consecutive sampling points and can be calculated by Eq (1). Velocity is calculated by taking the first-order derivative of displacement. Acceleration is the first-order derivative of velocity, the second-order derivative of displacement. Then, the jerk corresponds to the third-order derivative of displacement. Additionally, vertical and horizontal components of displacement, velocity, acceleration, and jerk are computed to demonstrate characteristics in different directions. Vertical (or horizontal) displacement can be obtained as the vertical (or horizontal) distance between two consecutive sampling points. Similarly, the vertical or horizontal components of other features can be derived in the same way as described above. Pressure features include pressure and its first derivative, illustrating pressure changes over time.

$$\begin{array}{c} d_{i}=\{\begin{array}{cc} \sqrt{{\left(x_{i}-x_{i-1}\right)}^{2}+{\left(y_{i}-y_{i-1}\right)}^{2}}, & i=2,\cdots ,N\\ 0, & i=1 \end{array} \end{array}$$ (1)

Where *x*_*i*_ and *x*_*i*−1_ are the x-coordinates of the *i*th and (*i* − 1)th sampling points respectively, *y*_*i*_ and *y*_*i*−1_ are the y-coordinates of the *i*th and (*i* − 1)th sampling points respectively, and *N* is the number of sampling points.

### Moment feature

Moment is a physical quantity that describes the force and perpendicular distance from the point of action of the force. When the pen tip contacts the paper on the digitizing tablet, it is assumed that the applied pressure generates a moment at the pen tip, causing it to rotate relative to the center of rotation. By controlling the magnitude and direction of this moment, patients can control the movement of the pen, thereby achieving different shapes and lines in handwriting. Therefore, the magnitude of the moment is used as a dynamic feature to analyze handwriting signal, where moment equals the product of force magnitude and the vertical distance from the line of force to the center of rotation.

**Fig 4:**
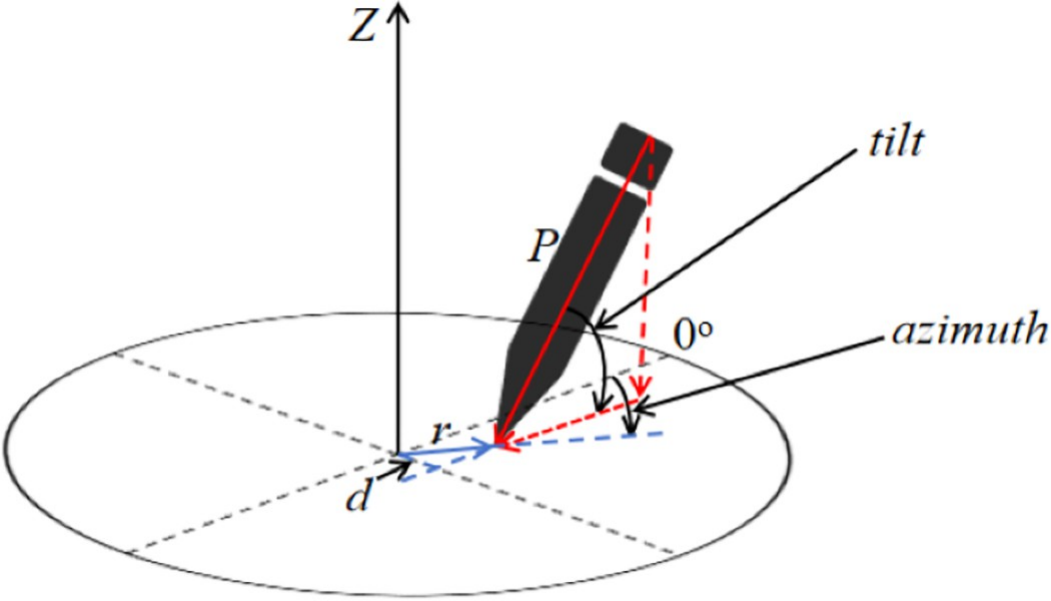
Handwritten schematic diagram.

Assuming pressure acts directly on the pen tip and considering only its effect, ignoring other external forces. The pen’s tilt angle *θ* and azimuth angle *φ* can be directly obtained from the raw signal. As shown in Fig 4, the component of pressure along the Z-axis direction does not generate a moment. A moment is only produced by components within the plane of rotation. Therefore, when calculating the moment, the component of pressure perpendicular to the Z-axis *P* × *cos*(*θ*) is used. To simplify calculations, the center of the handwriting is considered the center of rotation, which means all rotations are relative to the center of the handwriting. Thus, the distance from the point of pressure application to the center of rotation *r*, can be directly converted to the distance between the sampling point and the center of the handwriting. Therefore, its perpendicular distance *d* can be computed by *r* × *sin*(*φ*). Finally, the moment *M* is obtained as the product of the component of pressure perpendicular to the Z-axis and the vertical distance, as shown in Eq (2). The moment feature provides a new perspective for analyzing handwriting signals, comprehensively incorporating kinematic, pressure, and angle information.

$$\begin{array}{c} M_{i}=P_{i}\times \text{cos}\left(\theta_{i}\right)\times r_{i}\times \text{sin}\left(\varphi_{i}\right),\;i=1,2,3,\cdots ,N \end{array}$$ (2)

$$\begin{array}{c} r_{i}=\sqrt{{\left(x_{i}-\bar{x}\right)}^{2}+{\left(y_{i}-\bar{y}\right)}^{2}} \end{array}$$ (3)

Where $\bar{x}$ and $\bar{y}$ can be obtained by averaging the coordinates of all sampling points.

### Time-frequency-based statistical features exaction

Based on the dynamic handwriting signals, a feature extraction method is proposed to extract time-frequency-based statistical features of dynamic handwriting signals, aiming to thoroughgoing analyze the handwriting characteristics of PD or ET patients. It is noteworthy that all the features shown in Table 1 were used.

**Table 1:** Feature sets of different types.

Feature set	Feature	Description
Kinematic features	X	X-coordinate
	Y	Y-coordinate
	Displacement	Distance of pen moving at adjacent time points
	Velocity	Velocity of pen moving
	Acceleration	Acceleration of pen moving
	Jerk	Jerk of pen moving
	Displacement_h	Horizontal displacement component
	Velocity_h	Horizontal velocity component
	Acceleration_h	Horizontal acceleration component
	Jerk_h	Horizontal jerk component
	Displacement_v	Vertical displacement component
	Velocity_v	Vertical velocity component
	Acceleration_v	Vertical acceleration component
	Jerk_v	Vertical jerk component
Pressure features	Pressure	The pressure exerted
	Pressure_d	Pressure changes over time
Angle features	Tilt	Tilt angle
	Azimuth	Azimuth angle
Moment features	Moment	Moment generated under pressure

Firstly, for a single handwriting signal, compute 14 essential statistical functions: max, min, 1st quartile, 3rd quartile, interquartile range, 1st percentile, 99th percentile, percentile range, median, mean, standard deviation, variance, mean absolute deviation, and median absolute deviation. These features describe the degree of concentration and dispersion of the handwriting signal. Subsequently, to explore the distribution of the handwriting signal, the kurtosis, skewness, histogram and empirical distribution function of the handwriting signal are computed. The histogram’s vertical axis and the 20th and 80th percentiles as well as the slope of the empirical distribution function are also under consideration.

Furthermore, the autocorrelation, peaks, valleys, number of peaks, number of valleys, peak-to-peak value, traveled distance, and integrated trapezoidal rule-based area under the curve of the handwriting signal are computed in the temporal domain. Traveled distance is calculated from the distance between signal adjacent points. Simultaneously, entropy and multiscale entropy are used to describe the uncertainty of the handwriting signal. Next, to describe the symbol changes in the handwriting signal, the zero-crossing rate, number of local maximum/minimum values, number of direction changes, and relative direction changes are computed [42]. The differenced handwritten signals were used to characterize the degree of difference between adjacent sampling points of the handwritten signals. For the differenced signal *s*_*d*_ and its absolute value $s_{d}^{a}$, the median, mean, and sum were calculated to help understand the magnitude of variation and the degree of fluctuation of the handwritten signal.

Finally, Fast Fourier Transform (FFT) and Wavelet Transform (WT) are used. The frequency information of the handwriting signal in the frequency domain is revealed through FFT. Several features are extracted from the transformed handwriting signal to describe its frequency distribution, components, and characteristics. These features include mean coefficient, spectral distance, spectral fundamental frequency, spectral centroid frequency, spectral maximum frequency, and spectral maximum peak value features [43]. WT is used to characterize the signal’s energy distribution and waveform changes. Entropy, absolute mean, variance and standard deviation are extracted from the transformed handwriting signal to describe its overall energy and variability. Additionally, Mel Frequency Cepstral Coefficients (MFCC) are extracted.

### Feature selection

The number of features extracted from the original signal far exceeds the number of samples, leading to the problem of high-dimensional feature space. Therefore, feature selection methods are used to filter out the most representative and relevant subsets of features, reducing the dimension of the feature space. Specifically, four types of features are extracted: kinematic features, pressure features, angle features, and moment features. Due to their different physical meanings and statistical properties, feature selection is separately used for each type. The Random Forest feature selection method is used to select the top 30 features based on their importance rankings for each type of feature. In particular, this article details the reasons for choosing the random forest feature selection method in Selection of feature selection methods and classifiers. Through this approach, not only can the dimension of the feature space be reduced, but also ensures that the selected feature subset is highly representative and relevant.

### The proposed eCOA algorithm

The COA algorithm is an optimization algorithm simulating the coatis’ natural behavior. COA consists of two phases: the exploration phase, which mimics the coati’s behavior of attacking iguanas, and the exploitation phase, which mimics the coati’s behavior of escaping predators. COA can search the space well and find better results. However, in some optimization situations, COA may lead to premature convergence. Therefore, the eCOA algorithm has been introduced to mitigate this issue. By providing different strategies based on the different escape probabilities of prey, the eCOA algorithm is more flexible and better in exploring the search space. This helps the algorithm to escape local optima and discover global optima more effectively.

#### eCOA’s initialization phase

During the initialization phase, similar to other optimization algorithms, the eCOA algorithm randomly initializes the positions of coatis. The initialization process is shown as Eq (4).

$$\begin{array}{c} \begin{array}{c} X_{i}:x_{i,j}=lb_{j}+r\cdot \left(ub_{j}-lb_{j}\right),\\ \;i=1,2,\cdots ,N,\;j=1,2,\cdots ,m \end{array} \end{array}$$ (4)

Where *X*_*i*_ denotes the location of the *i*th coati, *x*_*i*,*j*_ denotes the value of the *j*th dimension for the *i*th coati, *N* is the number of coatis, *m* is the number of dimensions, *r* is a random number which range from [0, 1], and *lb*_*j*_ and *ub*_*j*_ are the lower and upper bounds of the *j*th dimension, respectively.

#### eCOA’s exploration phase

In the exploration phase, the coati population is divided into two groups. One group is in trees, where the position of iguanas *Iguana* is considered the optimal location. The other group is on the ground, waiting for iguanas to fall to the ground, surrounding and hunting them. The position of iguanas *Iguana* is represented in Eq (5).

$$\begin{array}{c} \begin{array}{cc} \text{Iguana:}\;{\text{Iguana}}_{j} & =\{\begin{array}{cc} x_{j}^{\text{best}}, & i\geq \frac{N}{2}\\ lb_{j}+r\cdot \left(ub_{j}-lb_{j}\right), & \text{else} \end{array},\\ \;j & =1,2,\cdots ,m \end{array} \end{array}$$ (5)

Where $x_{j}^{best}$ represents the value of the *j*th dimension at the best position, and *Iguana*_*j*_ represents the value of the *j*th dimension for iguanas.

Due to the possibility of COA falling into local optima in some optimization problems, an escape probability for iguanas is considered in the original COA algorithm. Let *r*_*escape*_ denote the probability of iguanas escaping. When *r*_*escape*_ < 0.5, iguanas have a lower probability of successfully escaping, coatis adopt a soft encirclement strategy to trap iguanas, thus relaxing the restriction on the search space boundary to some extent. At this point, the position of the coatis is modeled using Eq (6). After determining the new movement position *Y*_*i*_, they evaluate the effectiveness of this move $F_{i}^{Y}$ to assess its efficiency. If the results are unfavorable (such as iguanas displaying more cunning escape strategies), coatis quickly adjust their approach and begin implementing a series of new movements to capture the prey. Considering the irregularity and rapidity of the Levy flight [44], this paper assumes that the coatis move according to the Levy flight mode, as shown in Eq (7). This helps the coatis better adapt to the prey’s escape behavior and thus increases the probability of a successful capture.

$$\begin{array}{c} Y_{i}:y_{i,j}=Iguana_{j}-r\cdot \left|2\cdot \left(1-r\right)\cdot Iguana_{j}-x_{i,j}\right| \end{array}$$ (6)

$$\begin{array}{c} X_{i}^{P1}:x_{i,j}^{P1}=\{\begin{array}{cc} y_{i,j}, & F_{i}^{Y}<F_{i},\\ y_{i,j}+r\cdot LF, & \text{else}, \end{array} \end{array}$$ (7)

When *r*_*escape*_ ≥ 0.5, iguanas have a greater probability of successfully escaping, coatis employ a hard encirclement strategy to trap iguanas, strictly controlling the search space boundaries. At this time, the position of coatis is given by Eq (8).

$$\begin{array}{c} X_{i}^{P1}:x_{i,j}^{P1}=Iguana_{j}-r\cdot \left|Iguana_{j}-x_{i,j}\right| \end{array}$$ (8)

If the updated new position improves the objective function value, the update process continues. Otherwise, the coatis remain in their current positions. This update process is modeled with Eq (9).

$$\begin{array}{c} X_{i}=\{\begin{array}{cc} X_{i}^{P1}, & F_{i}^{P1}<F_{i},\\ X_{i}, & \text{else}, \end{array} \end{array}$$ (9)

Where *X*_*i*_ is the updated position of the *i*th coati, $X_{i}^{P1}$ denotes its new position, $X_{i,j}^{P1}$ represents its new value on the *j*th dimension, $F_{i}^{P1}$ is its new objective function value, and *F*_*i*_ is its original objective function value. *LF* follows a Levy flight distribution.

#### eCOA’s exploitation phase

During the exploitation phase, the coatis encounters an attack and escapes from its current location to a safe location nearby. This behavior is modeled with Eq (11).

$$\begin{array}{c} lb_{j}^{local}=\frac{lb_{j}}{t},\;ub_{j}^{local}=\frac{ub_{j}}{t},\;t=1,2,\cdots ,T. \end{array}$$ (10)

$$\begin{array}{c} \begin{array}{cc} X_{i}^{P2}:x_{i,j}^{P2} & =x_{i,j}+\left(1-2r\right)\cdot \left(lb_{j}^{local}+r\cdot \left(ub_{j}^{local}-lb_{j}^{local}\right)\right),\\ \;i & =1,2,\cdots ,N,\;j=1,2,\cdots ,m, \end{array} \end{array}$$ (11)

Where *t* is an iteration counter and *T* is the total number of iterations.

Similar to the exploration phase, if the update improves the objective function value, the update is accepted. Otherwise, the position remains unchanged. This update process is modeled using Eq (12).

$$\begin{array}{c} X_{i}=\{\begin{array}{cc} X_{i}^{P2}, & F_{i}^{P2}<F_{i},\\ X_{i}, & \text{else}. \end{array} \end{array}$$ (12)

#### eCOA’s termination phase

**Fig 5:**
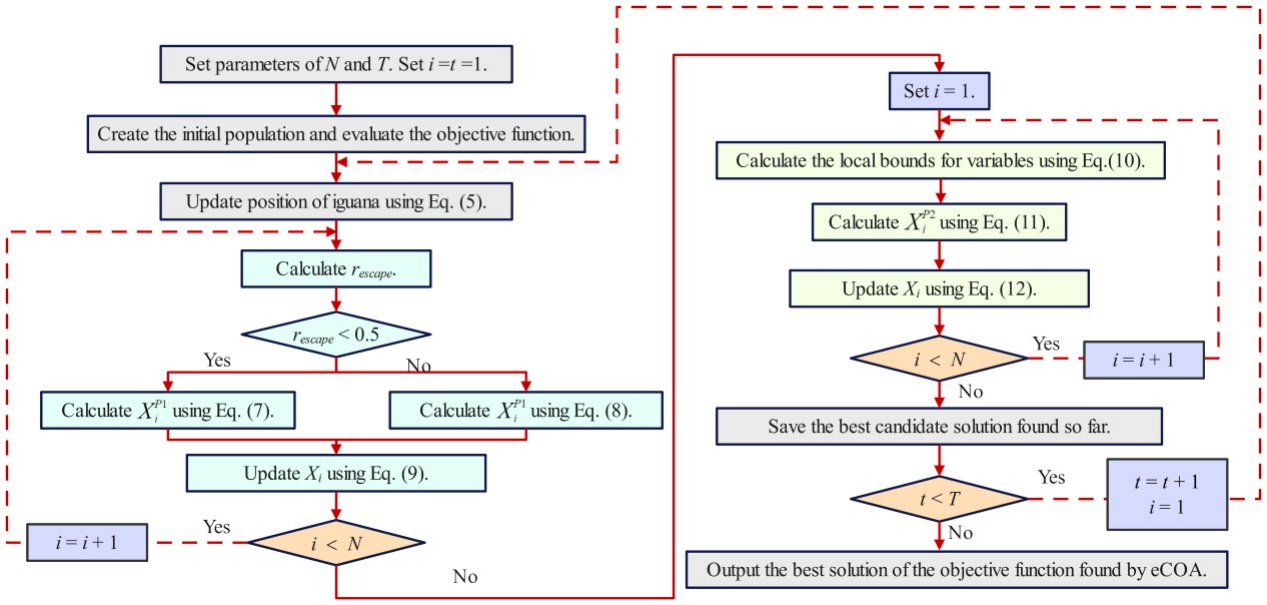
The flowchart of eCOA.

An iterative process is completed through Eqs (4) to (12). eCOA continues until the maximum number of iterations is reached and the best result is returned. The pseudo-code and flowchart of eCOA are illustrated in Algorithm 1 and Fig 5 respectively.

**Algorithm 1**. eCOA’s Pseudo-code.

**Start eCOA**.

**Input**: Objective function, population size *N*, number of dimensions, maximum iterations max_iter.

**Initialize** population by Eq (4) and evaluate fitness.

For *t* = 1 to *max_iter*

 Update location of the iguana by Eq (5)

 **eCOA’s exploration Phase**

 For *i* = 1 to *N*

  **If** (*r*_*escape*_ < 0.5) **then**

   Calculate the *i*th coati’s new position by Eq (7).

  **Else then**

   Calculate the *i*th coati’s new position by Eq (8).

  Update location of the *i*th coati by Eq (9).

 End for

 **eCOA’s exploitation Phase**

 Calculate the local bounds for each dimension by Eq (10).

 For *i* = 1 to *N*

  Calculate the *i*th coati’s new position by Eq (11).

  Update location of the *i*th coati by Eq (12).

 End for

 Save the best candidate solution found so far.

End for

**Output**: Best solution found.

**End eCOA**.

### eCOA-Adaboost classifier

An decision trees estimators based AdaBoost classifier is selected for PD detection, the details is discussed in Selection of feature selection methods and classifiers. It is crucial to choose classifier hyperparameters reasonably to improve prediction accuracy. In AdaBoost, four key hyperparameters need to be optimized: maximum tree depth *d*, minimum number of samples required to split a node *m*, number of base estimators *n*, and learning rate *r*. The eCOA algorithm was chosen to search for the optimal hyperparameters of the AdaBoost classifier. The prediction accuracy is the objective function and maximizes during the search process. The search process terminates upon reaching the maximum number of iterations. Specific steps can be formulated as: (1) Parameters and initial population positions are initialized, including *d* with a search range of [1, 30], *m* with a search range of [*e*^−4^,1], *n* with a search range of [1, 100], and *r* with a search range of [*e*^−6^,10]; (2) The eCOA algorithm iteratively updates individual positions to determine the optimal configuration of AdaBoost parameters; (3) The optimal parameter AdaBoost classifier is constructed.

Four types of features are individually inputted into the eCOA-AdaBoost classifier to obtain prediction probabilities. After receiving the prediction results, an adaptive weighted sum method is used to get the final prediction result for each sample. Specifically, each feature type is separately trained and predicted using the eCOA-AdaBoost classifier, resulting in prediction probabilities for each sample across different classes. For instance, for a sample *i*, the prediction probability under feature *k* is $P_{i}^{k}$. Then, the weights of the features are determined by the ratio of the number of predictions in their prediction category to the total number of predictions. Finally, the weighted sum of feature prediction probabilities yields the final prediction probability *P*_*i*_. The predicted category for sample *i* is the one with the highest predicted probability, as shown in Eq (13).

$$\begin{array}{c} P_{i}=\sum_{k}\frac{n_{i}^{c}}{N_{k}}P_{i}^{k} \end{array}$$ (13)

Where $w_{i}^{k}$ is the weight of the feature *k* when predicting the sample *i*. $n_{i}^{c}$ is the number of features whose prediction classes are the same as the feature *k* for the sample *i*, and *N*_*k*_ is the total number of all features used for prediction.

### Evaluation metrics

Several metrics are used to measure the performance of the proposed methods, including Accuracy (Acc), Specificity (Spe), Sensitivity (Sen), and AUC, defined in Eqs (14) to (16). Accuracy refers to the ratio of correctly predicted patients to the total number of patients. Taking PD as an example, Specificity indicates the probability of correctly predicting non-PD patients among all non-PD cases. Sensitivity indicates the probability of correctly predicting PD patients among all PD cases.

$$\begin{array}{c} Acc=\frac{TP+TN}{TP+TN+FP+FN} \end{array}$$ (14)

$$\begin{array}{c} Spe=\frac{TN}{TN+FP} \end{array}$$ (15)

$$\begin{array}{c} Sen=\frac{TP}{TP+FN} \end{array}$$ (16)

Where TP, FP, TN, and FN are true positive, false positive, true negative, and false negative, respectively.

The Receiver Operating Characteristic (ROC) curve is generated by plotting the true positive rate (TPR) and false positive rate (FPR) of classification results. TPR and FPR are calculated as shown in Eq (17). The Area Under the Curve (AUC) is the area enclosed by the ROC curve and the coordinate axes below it. It is noteworthy that a one-vs-one strategy is used to handle multiclass problems.

$$\begin{array}{c} TPR=\frac{TP}{TP+FN},\;FPR=\frac{FP}{TN+FP} \end{array}$$ (17)

## Results and discussion

In the experimental setup, the framework was implemented with Python 3.9 and Sklearn 1.3.2. The experiments were conducted on the Intel(R) Core(TM) i7-12700F, 2.10 GHz CPU. To objectively validate the effectiveness of the proposed framework, all experiments employed five-fold cross-validation.

### Selection of feature selection methods and classifiers

**Fig 6:**
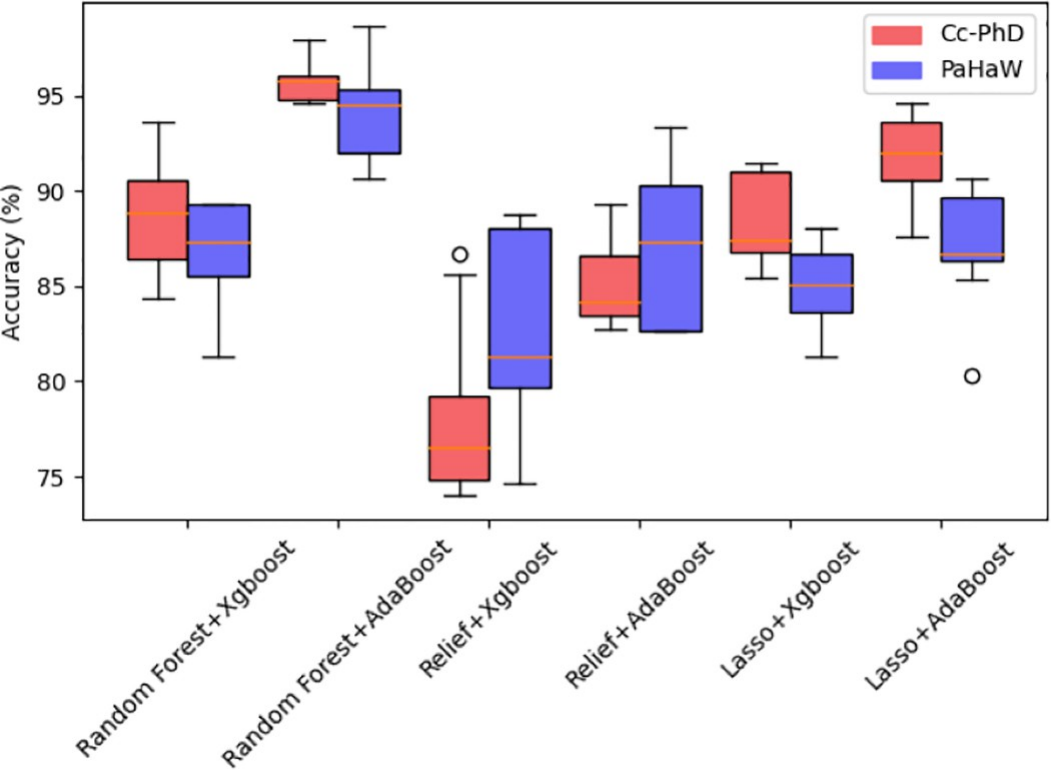
Box plot of accuracy in different tasks under different feature selection methods and classifiers.

This section explores several feature selection methods and classifier combinations to determine the most suitable one. Three feature selection methods: Random Forest, Relief, and Lasso are used. At the same time, two classic ensemble learning algorithms, AdaBoost and XGBoost, are chosen as classifiers. For the XGBoost classifier, three key parameters need to be optimized: maximum tree depth for tree splitting, number of base estimators, and learning rate. The upper and lower limit of three key parameters’ are consistent with AdaBoost’s. Based on different feature selection methods and classifier combinations, the accuracy is presented in Table 2. From Table 2, there are significant differences in performance across different tasks for various combinations of feature selection methods and classifiers. The combination of the Random Forest feature selection method and AdaBoost classifier reported the highest accuracy across all handwriting tasks. Additionally, Fig 6 shows that this combination not only achieves the highest average accuracy across different handwriting tasks but also exhibits less fluctuation between tasks. This may be attributed to the Random Forest method’s effective handling of high-dimensional data and feature correlation issues, along with AdaBoost’s ability to distinguish misclassified samples. Overall, the combination of these two methods can fully leverage their respective strengths to achieve the highest accuracy and stability. Therefore, Random Forest and AdaBoost were ultimately chosen as the framework’s feature selection method and classifier.

**Table 2:** Analysis of different feature selection methods and classifiers.

Dataset	Feature selection	Classifier	Task							
			1	2	3	4	5	6	7	8
Cc-PhD	Random Forest	Xgboost	84.63	91.74	84.37	90.18	86.96	93.62	89.24	88.36
		AdaBoost	94.84	97.95	95.84	94.56	94.56	96.73	95.73	95.79
	Relief	Xgboost	86.68	85.63	74.95	77.13	74.03	76.49	74.39	76.61
		AdaBoost	83.68	88.84	84.26	85.85	82.69	84.09	82.92	89.30
	Lasso	Xgboost	90.79	87.74	85.42	87.02	85.91	91.46	91.46	87.13
		AdaBoost	87.74	93.90	87.58	93.51	91.46	94.62	92.57	91.46
PaHaW	Random Forest	Xgboost	82.00	89.33	86.67	89.33	81.33	86.67	88.00	89.33
		AdaBoost	94.38	94.67	94.67	90.67	92.00	97.33	92.00	98.67
	Relief	Xgboost	88.76	74.67	82.67	78.67	88.00	80.00	88.00	80.00
		AdaBoost	90.19	82.67	93.33	90.67	88.00	86.67	82.67	82.67
	Lasso	Xgboost	86.10	86.67	88.00	84.00	84.00	81.33	82.67	86.67
		AdaBoost	80.29	89.33	90.67	86.67	86.67	85.33	90.67	86.67

### Statistical analysis

Two non-parametric tests were used to see the significance of the proposed features across groups in different handwriting tasks and dynamic handwriting signals: the Mann-Whitney U test and the Kruskal-Wallis test. These tests examine whether there are differences in features between different groups by testing if the medians of the groups are equal. The Mann-Whitney U-test is suitable for testing two independent samples, whereas the Kruskal-Wallis test is suitable for three or more independent samples. Therefore, the Mann-Whitney U test was applied to the PaHaW dataset, while the Kruskal-Wallis test was applied to the Cc-PhD dataset. Tables 3 and 4 show the p-values of the 10 most relevant features in each feature category, and the degree of relevance is calculated by Spearman’s correlation coefficient.

**Table 3:** Mann-Whitney U test with different features under different handwriting tasks on the PaHaW dataset. |*ρ*| is the Spearman’s correlation coefficient. Abbreviation: FMC: FFT mean coefficient, WAM: wavelet absolute mean.

Feature, functional, task number	|*ρ*|	P value	Feature, functional, task number	|*ρ*|	P value
Vertical, peaks_values_percentile_5, 8	0.52	2.64*10^-^^6^	Pressure_d, FMC_62, 2	0.39	5.63*10^-^^4^
Velocity, FMC_12, 3	0.52	2.98*10^-^^6^	Pressure, FMC_62, 2	0.37	1.13*10^-^^3^
Vertical, valleys_values_percentile_5, 8	0.52	4.00*10^-^^6^	Pressure_d, FMC_70, 2	0.35	2.09*10^-^^3^
Vertical, valleys_values_quartile_1, 8	0.51	5.67*10^-^^6^	Pressure_d, FMC_19, 1	0.35	2.86*10^-^^3^
Jerk_h, FMC_19, 3	0.51	6.73*10^-^^6^	Pressure, FMC_50, 3	0.35	2.34*10^-^^3^
Acceleration, FMC_10, 3	0.50	7.12*10^-^^6^	pressure_d, FMC_28, 2	0.34	3.02*10^-^^3^
Vertical, valleys_values_mean, 8	0.50	7.12*10^-^^6^	Pressure_d, FMC_20, 1	0.33	4.50*10^-^^3^
Jerk, FMC_11, 3	0.50	7.54*10^-^^6^	Pressure, FMC_28, 2	0.33	3.88*10^-^^3^
Acceleration, FMC_12, 3	0.50	7.98*10^-^^6^	Pressure_d, FMC_59, 2	0.33	3.88*10^-^^3^
Displacement, FMC_10, 3	0.50	7.98*10^-^^6^	Pressure, FMC_136, 2	0.33	4.17*10^-^^3^
Azimuth, FMC_53, 2	0.43	1.56*10^-^^4^	Moment, WAM_2, 2	0.39	5.87*10^-4^
Azimuth, FMC_1, 3	0.42	2.15*10^-^^4^	Moment, WAM_3, 2	0.38	7.84*10^-4^
Tilt, number of maximums, 1	0.39	1.03*10^-^^3^	Moment, WAM_1, 2	0.37	1.32*10^-^^3^
Tilt, FMC_21, 1	0.38	9.90*10^-^^4^	Moment, WAM_0, 2	0.34	2.71*10^-^^3^
Azimuth, FMC_123, 8	0.38	1.04*10^-^^3^	Moment, WAM_5, 2	0.34	2.92*10^-^^3^
Azimuth, MFCC_11, 4	0.37	1.08*10^-^^3^	Moment, WAM_4, 2	0.34	3.02*10^-^^3^
Tilt, FMC_18, 1	0.37	1.51*10^-^^3^	Moment, FMC_24, 2	0.33	3.88*10^-^^3^
Azimuth, FMC_74, 2	0.36	1.54*10^-^^3^	Moment, WAM_6, 2	0.32	6.08*10^-^^3^
Tilt, FMC_62, 7	0.36	1.60*10^-^^3^	Moment, FMC_48, 3	0.31	6.72*10^-^^3^
Azimuth, Wavelet entropy, 7	0.36	1.67*10^-^^3^	Moment, FMC_248, 8	0.31	7.42*10^-^^3^

**Table 4:** Kruskal-Wallis test with different features under different handwriting tasks on the Cc-PhD dataset. |*ρ*| is the Spearman’s correlation coefficient. Abbreviation: FMC: FFT mean coefficient, NCP: number_of_changes_in_pressure, rNCP: relative_number_of_changes_in_pressure.

Feature, functional, task number	|*ρ*|	P value	Feature, functional, task number	|*ρ*|	P value
Jerk_v, Histogram_0, 7	0.87	2.65*10^-^^16^	Pressure, MFCC_2, 7	0.40	6.19*10^-^^4^
X, Wavelet energy_1, 6	0.86	5.96*10^-^^16^	Pressure, Histogram_9, 7	0.37	2.57*10^-^^4^
X, Wavelet standard deviation_1, 6	0.86	5.96*10^-^^16^	Pressure, number of minimums, 2	0.37	9.10*10^-^^5^
X, Wavelet variance_1, 6	0.86	5.96*10^-^^16^	Pressure, NCP, 2	0.36	1.15*10^-^^4^
Jerk, Histogram_8, 5	0.86	1.23*10^-^^16^	Pressure, number of minimums, 1	0.36	1.50*10^-^^3^
X, Wavelet energy_2, 6	0.86	7.06*10^-^^16^	Pressure, rNCP, 2	0.36	1.60*10^-^^5^
X, Wavelet standard deviation_2, 6	0.86	7.06*10^-^^16^	Pressure, number of maximums, 2	0.35	2.17*10^-^^4^
X, Wavelet variance_2, 6	0.86	7.06*10^-^^16^	Pressure, NCP, 1	0.35	2.05*10^-^^3^
Jerk_h, Histogram_1, 3	0.85	2.50*10^-^^16^	Pressure, rNCP, 1	0.35	7.45*10^-^^4^
Jerk, Histogram_9, 5	0.85	1.52*10^-^^15^	Pressure, number of maximums, 1	0.33	3.71*10^-^^3^
Tilt, Sum absolute diff, 1	0.50	1.35*10^-^^6^	Moment, Peak to peak value, 7	0.82	3.97*10^-^^15^
Tilt, FMC_4, 1	0.41	2.82*10^-^^4^	Moment, FMC_57, 2	0.80	3.02*10^-^^14^
Tilt, FMC_157, 1	0.40	2.02*10^-^^4^	Moment, FMC_43, 2	0.80	3.02*10^-^^14^
Tilt, FMC_105, 1	0.40	4.22*10^-^^4^	Moment, Percentile range, 7	0.80	1.41*10^-^^14^
Tilt, FMC_54, 1	0.37	6.30*10^-^^4^	Moment, Signal distance, 2	0.80	1.69*10^-^^14^
Tilt, FMC_106, 1	0.37	8.12*10^-^^4^	Moment, Sum absolute diff, 2	0.80	1.69*10^-^^14^
Tilt, FMC_3, 1	0.36	1.41*10^-^^3^	Moment, FMC_40, 2	0.79	5.49*10^-^^14^
Tilt, FMC_199, 1	0.35	1.74*10^-^^3^	Moment, FMC_249, 1	0.79	5.88*10^-^^14^
Tilt, FMC_55, 1	0.35	8.51*10^-^^4^	Moment, FMC_28, 2	0.79	9.78*10^-^^14^
Tilt, FMC_71, 1	0.34	3.20*10^-^^3^	Moment, FMC_36, 2	0.79	7.18*10^-^^14^

Firstly, it can be seen that the p-values of all features are less than 0.05, indicating that their inter-group differences are statistically significant, suggesting significant differences among groups for these features. Secondly, by comparing the absolute values of Spearman correlation coefficients, kinematic features outperform other features. This is because both Parkinson’s disease and essential tremor are common movement disorders that cause abnormalities in speed, smoothness, and acceleration among patients. Therefore, kinematic features are more discriminative compared to other types of features. Additionally, in the PaHaW dataset, single features in the moment category perform relatively poorly compared to features in other categories. In contrast, in the Cc-PhD dataset, moment features perform relatively well, apart from kinematic features. This indicates that PD and ET patients exhibit abnormalities in moment features, although moment features may tend to reflect abnormalities more in ET patients. Finally, it is evident that most of the displayed features are frequency domain features of dynamic handwriting signals. This suggests that features extracted from dynamic handwriting signals after FFT and WT show superior performance. FFT and WT can convert dynamic handwriting signals to the frequency domain, revealing frequency characteristics that are rarely discovered in the time domain. Furthermore, PD and ET exhibit tremor symptoms with only partial overlap in dominant frequencies. Therefore, these features are effective.

### Ablation study

#### Moment features

With/without moment features across different handwriting tasks, the framework’s performance is analyzed in this section, with specific results shown in Table 5. After incorporating moment features, the framework’s effectiveness has significantly improved, with a substantial increase in prediction accuracy. Most handwriting tasks have shown varying degrees of improvement in (average) sensitivity, (average) specificity, and (average) AUC values. Moment features integrate information from kinematic, pressure, and angle features, providing more discriminative features. This enables the framework to comprehensively capture handwriting characteristics, enhancing overall performance.

**Table 5:** Ablation study on moment features.

Dataset	Task	Features	Acc (%)	Sen (%)	Spe (%)	AUC (%)
Cc-PhD	1	Without moment features	89.79	91.08	95.29	93.18
		With moment features	94.84	94.86	97.34	96.10
	2	Without moment features	92.79	92.29	96.38	94.34
		With moment features	97.95	98.15	99.17	98.66
	3	Without moment features	87.63	87.96	93.74	90.85
		With moment features	95.84	96.03	98.05	97.04
	4	Without moment features	90.12	90.63	94.87	92.75
		With moment features	94.56	96.07	97.65	96.86
	5	Without moment features	89.18	87.88	93.61	90.74
		With moment features	94.56	94.75	97.37	96.06
	6	Without moment features	87.25	85.61	93.71	89.66
		With moment features	96.73	96.95	98.59	97.77
	7	Without moment features	89.35	86.94	93.92	90.43
		With moment features	95.73	95.56	97.52	96.54
	8	Without moment features	89.41	87.67	94.07	90.87
		With moment features	95.79	95.72	97.66	96.69
PaHaW	1	Without moment features	83.24	86.00	82.70	84.35
		With moment features	94.38	91.50	97.78	94.64
	2	Without moment features	89.33	88.06	90.65	89.35
		With moment features	94.67	88.06	100.00	94.03
	3	Without moment features	88.00	88.61	89.49	89.05
		With moment features	94.67	97.50	91.52	94.51
	4	Without moment features	88.00	87.70	84.64	86.17
		With moment features	90.67	87.70	95.00	91.35
	5	Without moment features	89.33	90.00	84.49	87.25
		With moment features	92.00	87.78	94.85	91.31
	6	Without moment features	89.33	90.56	88.03	89.29
		With moment features	97.33	94.64	100.00	97.32
	7	Without moment features	86.67	83.25	92.35	87.80
		With moment features	92.00	95.28	89.02	92.14
	8	Without moment features	90.67	88.89	96.36	92.63
		With moment features	98.67	97.78	100.00	98.89

Specifically, on the Cc-PhD dataset, the add of moment features has improved performance prominently across all tasks. This indicates that moment features help the framework better distinguish between PD patients, ET patients, and HC, reducing the probability of misdiagnosis and underdiagnosis. On the PaHaW dataset, for tasks 1, 6, and 8, moment features have enhanced the framework’s ability to differentiate between PD patients and HC. For tasks 2, 3, 4, and 5, moment features have increased the framework’s attentive to features specific to HC, improving specificity and reducing the likelihood of misdiagnosing as PD. For task 7, the add of moment features has made the framework more sensitive in identifying PD patients, capturing more PD-specific characteristics, thereby improving sensitivity and reducing the likelihood of missed diagnosis.

#### Time-frequency-based statistical features

**Fig 7:**
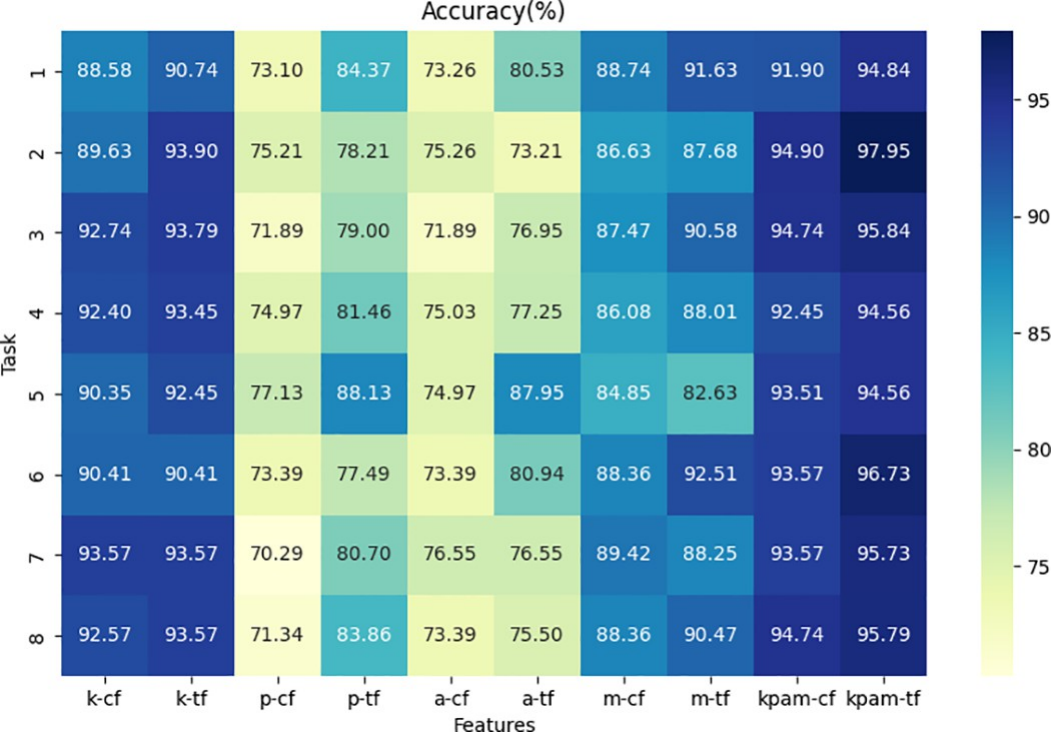
Accuracy scores based on different feature combinations in the Cc-PhD dataset. Abbreviations: a-b represents the extraction of feature b from dynamic feature data a. Features include: k (kinematic), p (pressure), a (angle), m (moment), kpam (kinematic, pressure, angle, and moment), cf (common features), and tf (TF-ST features).

**Fig 8:**
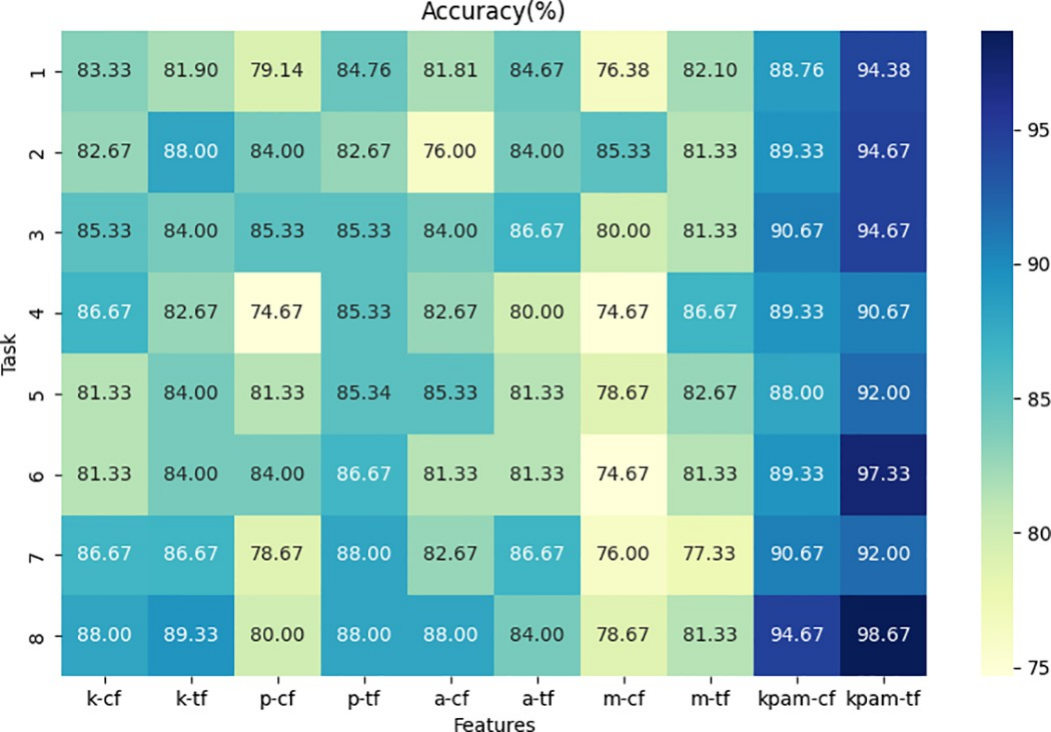
Accuracy scores based on different feature combinations in the PaHaW dataset. Abbreviations: a-b represents the extraction of feature b from dynamic feature data a. Features include: k (kinematic), p (pressure), a (angle), m (moment), kpam (kinematic, pressure, angle, and moment), cf (common features), and tf (TF-ST features).

This section analyzed the performance of the framework on various handwriting tasks under different types of features of the TF-ST features. Specific results can be found in Figs 7 and 8. The classification performance using TF-ST features was compared with a few widely used features. These commonly used features include basic statistical functions and some composite features, namely mean, median, maximum value, minimum value, standard deviation, first quartile, third quartile, interquartile range, first percentile, 99th percentile, percentile range, peaks, valleys, direction change count, and relative direction change count [17].

Firstly, the use of TF-ST features generally improved performance across most tasks and feature types. Integrating statistical features from the temporal-frequency domain provided the framework with multiple perspectives, aiding in better differentiation between PD patients, ET patients, and HC. We also found a few tasks where this approach did not show clear advantages. For example, angle features for task 2 and moment features for task 5 in the Cc-PhD dataset, etc., and kinematic features for task 1 and angle features for task 5 in the PaHaW dataset. This could be attributed to inter-feature correlations or redundant information in the tasks, where the feature selection method might not effectively filter out the most informative features, thereby leading to a slightly decrease in performance.

Next, compare the performance of frameworks using single-type features versus multiple types of features. When using the same features, integrating multiple types of features achieved superior performance across all tasks. This is because using only single-type features provides insufficient information to the framework, which can only discriminate based on single aspect of the features. Therefore, integrating multiple types of features helps the framework better identify PD patients, ET patients, and HC.

#### eCOA

This section presents the effectiveness of the eCOA algorithm to find optimal parameters and improve framework classification performance. The eCOA algorithm is compared with six metaheuristic algorithms: Grey Wolf Optimizer (GWO) [45], Harris Hawk Optimization (HHO) [44], Marine Predators Algorithm (MPA) [46], Reptile Search Algorithm (RSA) [47], White Shark Optimizer (WSO) [48], and COA. All algorithms use 50 populations and 10 maximum iterations. The control parameters for each compared algorithm are shown in Table 6. In addition, we also compute the average rankings and overall rankings (based on average rankings) of each algorithm across different metrics. This allows for a more intuitive comparison of the combined performance of the algorithms based on the rankings.

**Table 6:** Parameter values of the compared algorithms.

Algorithm	Parameter	Value
GWO	Convergence parameter (a)	Decreases linearly from 2 to 0.
MPA	Fish Aggregating Devices (FADs)	0.2
	Constant number	0.5
	Random vector	[0, 1]
	Binary vector	0 or 1
RSA	Sensitive parameter (*α*)	0.1
	Sensitive parameter (*β*)	0.005
	Evolutionary Sense (ES)	randomly decreasing values between 2 and -2
WSO	*f*_*m*_ *in*	0.07
	*f*_*m*_ *ax*	0.75
	a0	6.25
	a1	100
	a2	0.0005
	*τ*	4.11

**Fig 9:**
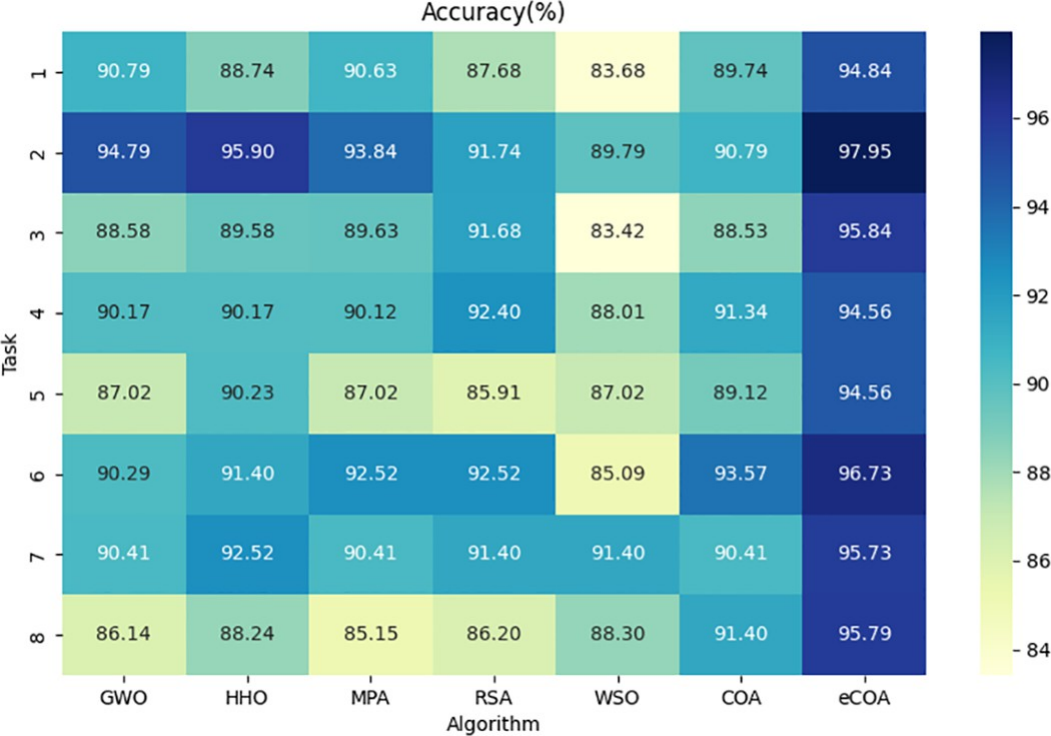
Accuracy score based on different optimization algorithms on the Cc-PhD dataset.

**Fig 10:**
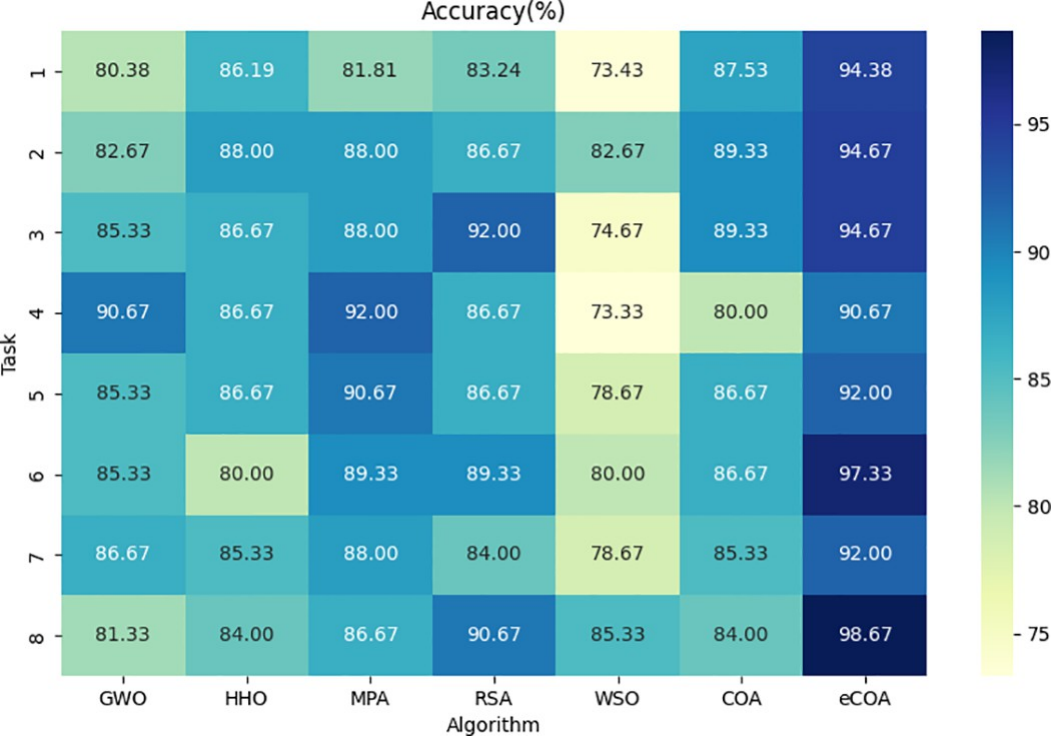
Accuracy score based on different optimization algorithms on the PaHaW dataset.

Table 7 shows the classification performance rankings under different optimization algorithms on the Cc-PhD and PaHaW datasets. On the Cc-PhD dataset, the eCOA algorithm ranked top in accuracy, sensitivity, specificity, and AUC scores across all tasks. On the PaHaW dataset, the eCOA algorithm achieves the same excellent results. Specifically, the eCOA algorithm achieved the best performance in most handwriting tasks, indicating its stability and reliability across different handwriting tasks. Based on the original COA algorithm, the eCOA algorithm enhances search capability by considering prey escape probabilities, enabling more effective exploration of parameter space to find optimal framework settings and improve performance. This improvement enhances the algorithm’s global search capability and increases its robustness and stability in complex classification tasks. Figs 9 and 10 show the accuracy scores based on different optimization algorithms.

**Table 7:** Performance comparison of different optimization algorithms for global optimization.

Dataset	Rank	Metric	Algorithm						
			GWO	HHO	MPA	RSA	WSO	COA	eCOA
Cc-PhD	Mean Rank	ACC	4.375	3.5	4.5	4.125	5.875	4	1
		Sen	5.25	3	4.75	4.25	5.75	4	1
		Spe	4.75	3.625	4.75	4.25	5.75	3.875	1
		AUC	5.25	3.25	4.625	4.25	5.625	4	1
	Total Rank	ACC	5	2	4	3	3	2	1
		Sen	6	2	4	3	3	2	1
		Spe	5	2	4	3	3	2	1
		AUC	6	2	4	3	3	2	1
PaHaW	Mean Rank	ACC	5.25	4.25	2.88	3.5	6.5	3.88	1.13
		Sen	4.75	3.38	2.88	2.63	6.38	5.25	2
		Spe	4.38	5.5	4	3.63	5.63	3.5	1.25
		AUC	5	4.5	2.88	3.5	6.5	4.25	1.38
	Total Rank	ACC	6	5	2	3	7	4	1
		Sen	5	4	3	2	7	6	1
		Spe	5	6	4	3	7	2	1
		AUC	6	5	2	3	7	4	1

### Comparison with State-of-the-art Methods

To further evaluate the proposed method’s effectiveness, we compared it with the baseline and state-of-the-art methods, and the results are displayed in Tables 8 and 9. Different studies are considered, including those using handwriting images or signals, as well as researches extracting handcrafted or deep features. Since these studies used different classifiers, data formats, and handwriting tasks, and reported varying classification performance, only the best results are chosen to compare in a fair manner.

**Table 8:** Performance comparison of different models on the PaHaW dataset.

Author(s)	Model	Features	Acc (%)	Sen (%)	Spe (%)	AUC (%)	Year
Drotár et al.	SVM	Kinematic and pressure features	81.3	87.4	80.9	-	2016
Kamran et al.	AlexNet	Image	62.5	-	-	-	2021
Diaz et al.	CNN+BiGRU	Kinematic and pressure features	96.25	92.5	100	96.88	2021
Valla et al.	LR	Angle-type and integral-like features	84.86	80.36	88.57	-	2022
Deharab et al.	SVM	AASR, ASODP	83.94	85.95	83.57	-	2022
Wang et al.	3D CNN	3D CNN-extracted	84.67	87.31	85.5	-	2024
Wang et al.	LSTM-CNN	Dynamic handwriting patterns	90.7	-	-	-	2024
Ours	Adaboost	Kinematic, pressure, moment and angle features	98.67	97.78	100	98.82	-

**Table 9:** Performance comparison of different models on the Cc-PhD dataset.

Author(s)	Model	Features	Acc (%)	Sen (%)	Spe (%)	AUC (%)	Year
Kamran et al.	AlexNet	Image	59.74	55.95	78.81	67.38	2021
Diaz et al.	CNN+BiGRU	Kinematic and pressure features	89.3	86.66	94.56	90.61	2021
Valla et al.	LR	Angle-type and integral-like features	75.37	76.5	88.39	82.44	2022
Deharab et al.	SVM	AASR, ASODP	81.26	79.67	90.92	85.29	2022
Wang et al.	3D CNN	3D CNN-extracted	73.33	69.11	85.61	77.36	2024
Ours	Adaboost	Kinematic, pressure, moment and angle features	97.95	98.15	99.17	98.66	-

The first compared baseline is the dynamic feature set composed of kinematic and pressure features proposed by Drotár et al. [17] The second baseline chosen is the image deep features extracted using a pre-trained AlexNet model proposed by Kamran et al. [49] Additionally, recent state-of-the-art works were selected for comparison. Diaz et al. [19] proposed a convolutional-BiGRU model to extract deep features from kinematic and pressure feature vectors. Valla et al. [10] added higher-order derivatives of kinematic and pressure feature vectors over time, including fourth-, fifth-, and sixth-order. Deharab et al. [11] proposed two graphical representation features of kinematic and pressure features. Wang et al. [21] encoded one-dimensional dynamic handwriting into three-dimensional voxelized point clouds and used a three-dimensional CNN model to extract deep features. Wang et al. [22] extracted deep features based on localized handwriting segments by the proposed LSTM-CNN.

Since the Cc-PhD dataset contains three classes and the methods proposed by Drotár et al. and Wang et al. contain parts that are only applicable to dichotomization, these two methods were not considered in the Cc-PhD dataset.

From Tables 8 and 9, the proposed method achieved the best results compared to other methods. On the PaHaW dataset, the framework achieved the best classification performance in Task 8: 98.67% accuracy, 97.78% sensitivity, 100% specificity, and 98.82% AUC. On the Cc-PhD dataset, the framework achieved the best classification performance in Task 2: 97.95% accuracy, 98.15% average sensitivity, 99.17% average specificity, and 98.66% average AUC. The addition of moment and TF-ST features provided more discriminative features for the framework, and the integration of eCOA helped the framework find parameters closer to optimal parameters, thereby improving the performance.

### Generalization analysis

**Fig 11:**
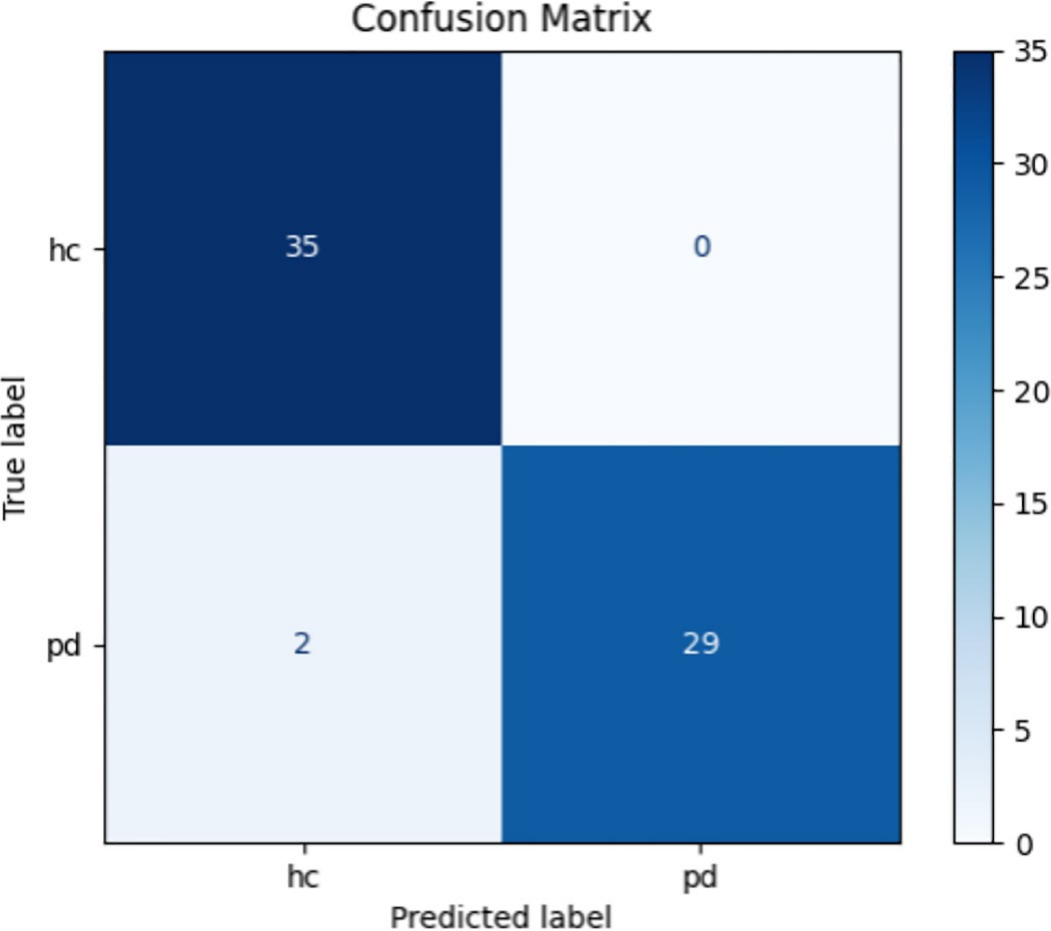
The confusion matrix of the provided method on the Archimedean spirals task.

To assess the framework’s generalization ability on real clinical data, cross-domain validation between the different datasets is conducted. We trained on the publicly available PaHaW dataset and tested on the self-built Cc-PhD dataset. The publicly available PaHaW dataset is used for training, and the self-constructed Cc-PhD dataset is used for validation. Since Archimedean spirals was chosen for both datasets, it was selected for the experiment, which is Task 1. Fig 11 displays the proposed method’s confusion matrix on the Cc-PhD dataset. It shows that there are no misclassifications among patients predicted as PD, with only a minimal number of PD patients not being identified. The framework achieved 96.97% accuracy, 100% specificity, 93.55% sensitivity, and 96.77% AUC. Overall, the framework effectively distinguishes PD patients from HC. This indicates that the framework performs strong generalization ability on external real clinical data and can be used to detect PD patients in real clinical applications.

## Conclusions

This paper proposes a feature extraction method based on handwriting for detecting PD. Specifically, building upon kinematic, pressure, and angle dynamic features, we propose a moment feature to integrate information from all three aspects. Subsequently, we propose a novel feature extraction method to capture a more comprehensive feature set by extracting TF-ST features from each dynamic feature. This results in a feature set encompassing time-frequency-based statistical features of dynamic handwriting signals’ kinematic, pressure, angle, and moment types. Then, we apply feature selection methods separately to each type of feature to select the most relevant features. Finally, machine learning classifiers are employed for PD detection. To enhance framework performance, we propose an eCOA algorithm to search for optimal framework parameters. Experimental results on the Cc-PhD and PaHaW datasets demonstrate accuracy rates of 97.95% and 98.67%, sensitivities of 98.15% (average) and 97.78%, specificities of 99.17% (average) and 100%, and AUCs of 98.66% (average) and 98.89%, respectively.

The proposed approach exhibits superior performance in PD detection compared to other methods. Firstly, the join of moment feature effectively integrates kinematic, pressure, and angle information to provide a novel perspective on describing dynamic handwriting signals. Secondly, extracting TF-ST features from all types of feature vectors enhances discriminative capability, aiding the framework in understanding differences between PD patients, ET patients, and HC from multiple perspectives. Lastly, by integrating the proposed eCOA algorithm, results closer to optimal parameters are achieved, thereby enhancing classification performance. Experimental results on the PaHaW and Cc-PhD datasets confirm the proposed method’s effectiveness. Moreover, generalization experiments demonstrate its applicability in real-world scenarios, supporting clinical practitioners in Parkinson’s disease detection.

Although our study has shown good results, it only included essential tremor, a disease easily confused with PD and did not consider disease subtypes and stages. However, besides essential tremor, there are other diseases that can also be confused with PD. Therefore, in future work, we will continue expanding and refining our sample range to include more diverse cases, better meeting clinical needs.
